# The cross-cultural adaptation, validity, and reliability of the Spanish version of the Fremantle Back Awareness Questionnaire

**DOI:** 10.3389/fpsyg.2023.1070411

**Published:** 2023-03-02

**Authors:** Nuria García-Dopico, Alejandro De La Torre-Luque, Benedict Martin Wand, Olga Velasco-Roldán, Carolina Sitges

**Affiliations:** ^1^University of the Balearic Islands (UIB), Research Institute of Health Sciences (IUNICS) and Balearic Islands Health Research Institute (IdISBa), Palma, Spain; ^2^Department and Faculty of Nursing and Physiotherapy, University of the Balearic Islands (UIB), Palma, Spain; ^3^Department of Legal Medicine, Psychiatry and Pathology, Faculty of Medicine, Complutense University of Madrid, Madrid, Spain; ^4^Faculty of Medicine, Nursing and Midwifery and Health Sciences, University of Notre Dame Australia, Fremantle, WA, Australia; ^5^Department and Faculty of Psychology, University of the Balearic Islands (UIB), Palma, Spain

**Keywords:** low back pain, body image, back awareness, proprioception, self-concept, validity, reliability

## Abstract

**Introduction:**

In chronic low back pain (CLBP), disturbed body image has been highlighted as a contributor to the condition and a potential target for treatment. The Fremantle Back Awareness Questionnaire (FreBAQ) allows its assessment. Following international guidelines for the cross-cultural translation of questionnaires, we aimed to translate the FreBAQ into Spanish (FreBAQ-S) and validate the new questionnaire in a sample of Spanish-speaking people with CLBP.

**Methods:**

Two hundred and sixty-four adults with CLBP (91 males) and 128 healthy controls (34 males) completed an online form including the FreBAQ-S and questionnaires related to the pain experience. All participants were Spanish and no gender identities differing from biological sex were reported. A week later, 113 CLBP participants and 45 healthy controls (41 and 13 males, respectively), re-answered the FreBAQ-S to evaluate test–retest reliability. Confirmatory factor and multigroup analysis assessed the scale consistency on the patient sample. Discriminant and convergent validity were explored by between-group differences and the relationship with clinical characteristics. Reliability relied on Cronbach’s alpha estimates and test–retest (intraclass correlation coefficient, standard error of measurement, minimal detectable change).

**Results and discussion:**

Confirmatory factor analysis showed a one-factor structure of the questionnaire, without supporting evidence for item deletion (CFI = 0.97; TLI = 0.96; RMSEA = 0.06; SRMR = 0.07; SRMRu = 0.064). Multigroup analyses do not support mean invariance between groups regarding health condition or sex. The FreBAQ-S demonstrated good discriminant and convergent validity, internal consistency (*α* = 0.82), and test–retest reliability (ICC = 0.78; SE = 3.41; MDC = 5.12). The FreBAQ-S is a valid and reliable tool to assess back awareness in clinical and non-clinical samples.

## 1. Introduction

Chronic low back pain (CLBP) is the leading cause of disability worldwide (Abbafati et al., 2020), and the lifetime prevalence has been estimated to be as high as 70% (Delitto et al., 2012). It is considered a growing public health problem that significantly affects quality of life and functional status (Alonso-García and Sarría-Santamera, 2020). Specifically, the prevalence of CLBP has increased by more than 50% since 1990 and is expected to continue increasing (Clark and Horton, 2018). Factors such as age, fitness level, weight, occupation, and socio-psychological factors have been related to CLBP appearance and chronification. It is also associated with high healthcare utilization, treatment costs, and significant loss of work productivity. In this regard, the cost of CLBP in Spain represents 0.68% of the Gross Domestic Product in terms of direct and indirect costs (Alonso-García and Sarría-Santamera, 2020). Thus, CLBP is a burden not just for individuals, but also for healthcare systems, economies, and society at large. Added to its high and increasing socioeconomic burden, current interventions based on a biomedical model appear to provide only small, short-term improvements in outcome. This has prompted calls for researchers to work to expand their understanding of the problem and identify new targets for intervention (Macfarlane et al., 2006; Costa et al., 2013). Particularly, approaches that reflect the complex interaction of factors that influence pain across the biopsychosocial spectrum (Rizzo et al., 2022).

Chronic low back pain is associated with several proprioceptive deficits (Lee et al., 2010), such as reduced motion detection, diminished repositioning accuracy and decreased balance ability, among others (Šarabon et al., 2021). From those, there is a growing interest in the role of disturbed body image, both as a contributor to persistent pain, and as a potential target for treatment (Lotze and Moseley, 2007; Moseley, 2008; Crowe et al., 2010; Wand et al., 2014, 2016). Body image, or how our body feels to us in terms of its size, shape, and distinctive features, is essential for our daily living (Lotze and Moseley, 2007; Themelis et al., 2022). Body image has been shown to be disrupted in CLBP. People with CLBP report an altered sense of ownership and awareness of their back (Birklein and Maihöfner, 2012; Moseley et al., 2012), distorted perceived back size and shape (Wand et al., 2014), perceptions of fragility and vulnerability of the back (Darlow et al., 2015), feelings of exclusion, alienation, and rejection of the painful area (Crowe et al., 2010) and distorted representation when asked to draw how the back feels to them (Moseley, 2008).

Body image is considered a complex concept involving aspects of cognition, affect, perception, and behavior (Esposito et al., 2018; Themelis et al., 2022). Thus, in the assessment of body image, it is also important to consider the presence of changes in the central nervous system, which have been well-documented in people suffering from CLBP (Nijs et al., 2017). With the rise of neurosciences, several studies support the presence of functional (Kregel et al., 2015), morphological (Wand et al., 2011; Kregel et al., 2015) and neurochemical changes (Zhao et al., 2016) in brain areas thought to sub-serve body image in people with CLBP. There is also evidence of disruption in some of the mechanisms that contribute to body image, such as reduced motor detection, diminished repositioning accuracy, decreased balance degraded processing of tactile stimuli (Moseley, 2008; Wand et al., 2010, 2013), impaired lumbopelvic motor control (Hodges et al., 2019), and impaired trunk motor imagery performance (Bray and Moseley, 2011; Bowering et al., 2014). However, the link between body image disruptions and those changes needs to be explored further.

As body perception is largely driven by sensory input from touch, vision, and proprioception relevant to the body part, from interoceptive signals, as well as through interactions with the external world (Themelis et al., 2022), the above-mentioned changes may be influencing not only body image, but also the pain experience itself. Thus, body image should be correctly assessed when assessing populations in pain. In this regard, a recent systematic review (Ehrenbrusthoff et al., 2018a) called for valid, reliable, and simple tools to assess body image disruption. To the best of our knowledge, there is currently only one instrument assessing this construct in people suffering from CLBP, which is the Fremantle Back Awareness Questionnaire (FreBAQ). The FreBAQ is a quick, low-cost tool that claims to assess back-specific altered body image in CLBP composed of nine items (Wand et al., 2014). The questionnaire has been cross-culturally adapted into several languages and the psychometric properties of the original (Wand et al., 2016) and translated versions (Janssens et al., 2017; Nishigami et al., 2018; Ehrenbrusthoff et al., 2018b; Erol et al., 2019; Mahmoudzadeh et al., 2020; Rao et al., 2021) seem acceptable. Moreover, FreBAQ scores have been consistently associated with clinical variables, such as pain intensity and duration, and catastrophizing, pointing to satisfactory levels of convergent validity (Janssens et al., 2017; Nishigami et al., 2018; Erol et al., 2019). Questionnaire scores have also been associated with disability, anxiety, depression, fear-avoidance beliefs, and fear of movement, though with inconsistencies across language-validated versions (Wand et al., 2016; Nishigami et al., 2018; Erol et al., 2019; Rao et al., 2021).

There is currently no Spanish version of the FreBAQ, which limits the assessment of this relevant variable. Furthermore, there is no previous information on how common altered body image is and how is it correlated with other typically pain-related variables in Spanish-speaking people with CLBP. As it was stated, the socioeconomic burden imposed by CLBP management in Spain is high, and novel assessment tools are needed to tackle the variables that could be associated with the pain experience. Thus, the aims of this study were to develop a Spanish version of the FreBAQ, the FreBAQ-S, and evaluate its psychometric properties in a group of Spanish-speaking people with CLBP. Based on previous evidence, we expected higher FreBAQ-S scores in participants with CLBP (Janssens et al., 2017; Ehrenbrusthoff et al., 2018b; Mahmoudzadeh et al., 2020), and these scores to be positively correlated with clinical status (Nishigami et al., 2018; Ehrenbrusthoff et al., 2018b; Erol et al., 2019).

## 2. Materials and methods

### 2.1. Study design

This observational, case–control, cross-cultural adaptation and validation study was performed with clinical and community samples from the Balearic Islands (Spain). The study received ethical approval from the Clinical Research Ethics Committee of the Balearic Islands (Spain, 4502/21 PI) and was conducted in accordance with the Declaration of Helsinki and the American Psychiatric Association (APA) ethical standards. All study participants provided written informed consent.

### 2.2. Participants

Participants were recruited through social media, posters on clinical and non-clinical settings, and institutional mailing. Initially, 463 people showed interest. After initial screening, *N* = 2 participants declined consent, *N* = 39 did not meet inclusion criteria, and *N* = 30 did meet any exclusion criterion and were, therefore, excluded (*N* = 71). All included participants were Spanish. No gender identities differing from biological sex were reported. A sample of adults with CLBP and a sample of healthy pain-free adults were recruited between the 29th April and the 10th October 2021.

#### 2.2.1. Chronic low back pain sample

We recruited 264 participants with CLBP. The inclusion criteria were age over 18, suffering from non-specific CLBP (>3 months), and being fluent in Spanish. Participants were excluded if pregnant or early post-partum, if they had a specific cause for their LBP (cancer, infection, nerve root pain, inflammatory condition, etc.), or if they presented with severe scoliosis; psychological illness (major depressive, generalized anxiety, psychotic, or bipolar disorder); a central neurological disorder; a terminal illness; substance dependence; criminal litigation; or financial compensation related to their pain problem.

#### 2.2.2. Control sample

We recruited 128 healthy participants. Following the protocol used in the original development paper (Wand et al., 2014), the inclusion criteria were age over 18, currently back pain-free, and no episode of back pain within the last 2 years restricting work or leisure activities. Healthy participants were also required to be fluent in Spanish. The exclusion criteria were the same as above.

### 2.3. Instruments

All participants completed the validated Spanish versions of the following questionnaires:

#### 2.3.1. Fremantle back awareness questionnaire

The scale measures back awareness in people with CLBP. It comprises 9 items with a five-point Likert scale, expressed as a total score out of a maximum of 36 points, assessing neglect-like symptoms (items 1–3), proprioceptive acuity (items 4, 5) and trunk shape and size (items 6–9) (Wand et al., 2014, 2016).

#### 2.3.2. Numerical pain rating scale for pain intensity

Participants were asked to rate their pain intensity on a numerical pain rating scale ranging from 0 (no pain) to 10 (worst imaginable pain). Separate ratings were taken for current pain, usual pain, and worst pain. The NPRS has been shown to have concurrent and predictive validity as a measure of pain intensity (Jensen and McFarland, 1993; Childs et al., 2005).

#### 2.3.3. Tampa scale for Kinesiophobia

The scale measures fear of movement or fear of re-injury during movement. It was first introduced in an unpublished report by Miller et al. (1991). The original version included 17 items, with 4 of them reverse scored (Vlaeyen et al., 1995). The Spanish version improved the psychometrics by removing 6 items, including the 4 reverse scored. Thus, the Spanish version of the TSK comprises 11 items with a four-point Likert scale with two factors: activity avoidance and harm. The score range is 11–44, with a higher value reflecting a higher degree of kinesiophobia. Cronbach alpha for the scale was 0.72 (Gómez-Pérez et al., 2011).

#### 2.3.4. Pain catastrophizing scale

This 13-item questionnaire measures pain catastrophizing with a 3-factor structure (rumination, magnification, and helplessness). The scale demonstrated appropriate internal consistency (*α* = 0.79) (Sullivan et al., 1995; García Campayo et al., 2008). A total score is yielded (ranging from 0 to 52), along with three subscale scores assessing rumination, magnification, and helplessness.

#### 2.3.5. Depression, anxiety, and stress scale

This 21-item questionnaire measures depression, anxiety, and stress with 3 factors named as the 3 assessed constructs. Cronbach alpha for the scale was between 0.7 and 0.84 (Bados et al., 2005; Osman et al., 2012). A total score is yielded (ranging from 0 to 63), along with three subscale scores assessing depression, anxiety, and stress.

#### 2.3.6. Fear-avoidance beliefs questionnaire

This 16-item questionnaire measures fear avoidance and beliefs about pain with a two-factor structure (work and physical activity). The score range is 0–96, with a higher value reflecting a higher degree of fear-avoidance beliefs (Waddell et al., 1993; Kovacs et al., 2006).

#### 2.3.7. Pain vigilance and awareness questionnaire

This 9-item scale measures excessive attention to pain with a two-factor solution assessing active vigilance and passive awareness. Cronbach’s alpha for the scale was 0.8 (McCracken, 1997; Roelofs et al., 2003; Esteve et al., 2013). The score range is 0–45, with a higher value reflecting a higher degree of pain vigilance and awareness.

#### 2.3.8. International physical activity questionnaire

This 7-item scale measures the time spent last week on sedentary activities, walking, and moderate and vigorous physical activity. Cronbach’s alpha for the scale was 0.8 (Hagströmer et al., 2006; Mantilla Toloza and Gómez-Conesa, 2007).

In addition, participants with CLBP completed three additional Spanish-version validated questionnaires:

#### 2.3.9. STarT back screening tool

This 9-item tool stratifies people with LBP into low medium or high risk of poor outcome and has been used to guide the provision of early secondary prevention in primary care for low back pain (Hill et al., 2008; Gusi et al., 2011). The score range is 0–9, with a higher value reflecting a worse prognosis for CLBP.

#### 2.3.10. Oswestry disability index

This 10-item questionnaire measures low back pain-related disability. The sum of the 10 scores is expressed as a percentage, ranging from 0% (no disability) to 100% (maximum disability) (Fairbank and Pynsent, 2000; Selva-Sevilla et al., 2019).

#### 2.3.11. Central sensitization inventory

This scale measures symptoms related to central sensitivity syndromes and central sensitization. The questionnaire consists of two parts. Part A includes 25 questions related to common central sensitization symptoms. The score range is 0 to 125, with a higher value reflecting a higher degree of central sensitization. Part B determines if the patient has been diagnosed with certain disorders related to central sensitization. The scale demonstrated high internal consistency (*α* = 0.8) (Mayer et al., 2012; Cuesta-Vargas et al., 2016).

### 2.4. Procedure

#### 2.4.1. Cross-cultural adaptation

To produce the Spanish version of the Fremantle Back Awareness Questionnaire (FreBAQ-S), a forward-backward translation process was used. In this process, usually, two bilingual researchers participate: one translating the questionnaire from the source language and the second blindly translating back from the target language to the source (Brislin, 1970). However, in the International Test Commission Guidelines for Translating and Adapting Tests (Second Edition) (2017), it is suggested to perform test adaptations with more than one researcher per language. If the two versions are identical, it is suggested that the target version from the middle of the process is equivalent to the source language forms (Brislin, 1970).

Following international guidelines for the transcultural adaptation of questionnaires (Beaton et al., 2000), two native Spanish speakers independently translated the FreBAQ into Spanish. One translator was a physical therapist well acquainted with the subject area, while the other one was a psychologist. After discussing wording discrepancies, a preliminary Spanish version was developed, and was independently back-translated by two native English speakers. Both native English translators were unfamiliar with the content of the questionnaire, and were not involved with pain assessment or management. Language inconsistencies were discussed by the four translators and with the developer of the original FreBAQ (BMW), who participated as an expert in identifying and solving any discrepancies found between the translations. Once consensus was achieved, we created the pre-final Spanish version of the FreBAQ-S. This version was tested in 28 volunteers with (*N* = 17, 7 males) and without (*N* = 11, 5 males) CLBP between 10th and 25th April 2021. Further information regarding this pilot testing can be found in Supplementary Material (Appendix A). Participants of both groups were asked to complete the FreBAQ-S and to comment on its acceptability, comprehensibility, and time burden. As no participant gave any suggestion on potential improvements, nor reported any problem regarding the abovementioned aspects, no issues emerged from this step. Thus, the final version of the 9-item FreBAQ-S was established. A copy can be found in Supplementary Material (Appendix B).

#### 2.4.2. Data acquisition

All the data reported in the present manuscript were gathered specifically for this study. Data were collected between 29th April and 10th October 2021. To protect the participants against SARS-CoV-2, and to comply with social contact limitations imposed in our country during that period, the data acquisition was performed *via* an on-line survey. To be accepted in the study, willing adult volunteers had to access the on-line form of the study. First, they were asked to read the information sheet of the study and then to declare informed consent before proceeding to eligibility screening. Those who met the inclusion criteria and did not meet any exclusion criteria were considered participants of the study and were able to continue with the online form. All participants provided sociodemographic (age, sex, height, and weight) and clinical data (pain duration, medication use, and previous surgeries). Participants next completed the FreBAQ-S and the validated Spanish versions of the questionnaires outlined above. All participants were sent an email 1 week later asking them to complete a second online survey. This second survey included the FreBAQ-S and the NPRS to enable the assessment of test–retest reliability.

### 2.5. Data analysis

Normality was assessed for all the variables, testing parametric and non-parametric statistics on the non-normal *a priori* variables. After inspection, parametric statistics were utilized. Descriptive statistics are provided by group, with contrast estimator and effect sizes. Differences between groups were assessed with Student’s *t*-test and Chi-square (*χ*^2^) test. Multivariate normality hypothesis could not be upheld, as Mardia’s skewness test (b1,2 = 16.33, *z* = 1067.19, *p* < 0.01) and kurtosis test (b2,2 = 122.8, *z* = 16.74, *p* < 0.01) were both significant. Data were analyzed using RStudio (version 4.1.1; packages: lavaan, psych, corrplot) (RStudio-Open Source and Professional Software for Data Science Teams, 2021).

#### 2.5.1. Evidence of validity

The validity of the FreBAQ-S was explored through one-factor Confirmatory Factor Analysis (CFA) on the CLBP sample, with Diagonally Weighted Least Squares (DWLS) methods. DWLS estimator was preferred because we were aware of the lack of multivariate normality and this method outperformed other estimators when multivariate normality could not be assumed (Li, 2016). In this regard, DWLS provides more accurate estimates than the usual maximum likelihood estimator, even under non-normally distributed data (Li, 2016). As previous research suggested the possibility of deleting item 9 (Wand et al., 2014; Janssens et al., 2017) and a feasible second dimension with items 4–6 (Wand et al., 2016; Janssens et al., 2017; Nishigami et al., 2018), the factor structure of an eight-item one-factor, two-correlated, and two-uncorrelated solutions was modeled and compared.

As suggested (Hu and Bentler, 1999), a “two criteria” strategy was followed. Thus, in addition to the exact fit of the model (*χ*^2^ value), we examined five additional fit indexes: the root mean square error of approximation index (RMSEA), the comparative fit index (CFI), the Tucker–Lewis index (TLI), and the root mean square of residuals (SRMR). Values of RMSEA < 0.05, CFI ≥ 0.95, TLI ≥ 0.95, and SRMR < 0.08 were used as indicative of good model fit for CFA (Hu and Bentler, 1999). Based on the recommendations of recent research (Maydeu-Olivares, 2017; Shi et al., 2020; Ximénez et al., 2022), we also provide the unbiased root mean square of residuals (SRMRu), as it has demonstrated its superiority to other fit indexes by enabling the assessment of the degree of a model misspecification across model size, sample size, and measurement quality (Ximénez et al., 2022). Multigroup factor analysis (MA) was performed under the tradition of measurement invariance (Meredith and Teresi, 2006), following a stepwise strategy comprising increasingly restrictive models. Model parameters were progressively constrained to be equal between groups. Group (CLBP vs. controls) and sex (men vs. women) were used as multigroup factors. To assess goodness-of-fit of MA, we used the *χ*^2^ statistic, the RMSEA with a 90% confidence interval (CI), the CFI, the TLI, and the SRMR. Multigroup effects were examined by comparing nested models, using the incremental RMSEA (ΔRMSEA), the incremental SRMR (ΔSRMR), and the incremental CFI (ΔCFI). Values of ΔCFI < −0.01 and ΔRMSEA > 0.015, or ΔSRMR > 0.03, support significant between-group differences between nested models (Meredith and Teresi, 2006). Discriminant validity was examined by comparing the FreBAQ-S scores between patients and controls using Student’s *t*-test (Wand et al., 2014). Floor and ceiling effects [> 15% of respondents achieving the higher or lowest scores (Ehrenbrusthoff et al., 2018b)] and item endorsement were explored in the CLBP group, considering endorsement of all responses different from “never” (see Supplementary Appendices C,D; Wand et al., 2014). Convergent validity was explored with univariate Pearson’s correlation, examining the relationships between the FreBAQ-S and the standardized questionnaire scores (Wand et al., 2014).

#### 2.5.2. Evidence of reliability

Reliability analysis was based on Cronbach’s alpha and test–retest, using intraclass correlation coefficient (ICC) with a 95% CI, standard error of measurement (SE), calculated as $\text{SD}\times \sqrt{1-\text{ICC}}$ , and minimal detectable change (MDC), defined as $1.96\times \sqrt{2\times \text{SE}}$ (Atkinson and Nevill, 1998; Janssens et al., 2017). An ICC ≥ 0.7 was indicative of acceptable test–retest reliability (Atkinson and Nevill, 1998).

## 3. Results

Participants’ data for the self-reported measurements are presented in Table 1. For further details regarding the history of pain and drugs consumption, (see Supplementary Appendices E,F, respectively). The CLBP group (*N* = 264) was, on average, 6.15 years older (*t* = 3.3; *p* < 0.001) and had a higher BMI (*t =* 2.37; *p* = 0.019) than the control group (*N* = 128). No significant differences were found for the remaining demographic variables.

**Table 1:** Sociodemographic, clinical, and self-reported data.

	CLBP (*n* = 264) Mean (SD)	HC (*n* = 128) Mean (SD)	Contrast test	Effect size
**Sex, male (N, %)**	91 (34.46)	34 (26.56)	4.4	0.07
**Age, years**	45.67 (11.59)	39.52 (12.66)	3.3^**^	0.5^††^
**Height, meters**	1.68 (0.1)	1.67 (0.08)	0.95	0.11
**Weight, kg**	74.5 (19.11)	65.57 (12.53)	2.58^**^	0.55^††^
**BMI**	25.23 (5.31)	23.42 (3.82)	2.37^**^	0.39^†^
**Working status**				
Active	213 (80.68)	114 (89.06)	3.79	0.07
On leave	16 (6.06)	0	6.61^*^	0.09
Retiree or pensioner	19 (7.19)	4 (3.12)	1.9	0.16
Unemployed	16 (6.06)	10 (7.81)	0.19	0.02
Months suffering pain	97.6 (103.29)	–	–	–
**Pain intensity** (Numerical Pain Rating Scale, 0–10), mean (SD)				
At the moment	5.28 (2.34)	0.57 (1.06)	17.92^**^	2.59^†††^
Usually	5.62 (2.01)	1 (1.36)	18.64^**^	2.69^†††^
At the worst moments	8.6 (1.29)	3.7 (2.77)	12.84^**^	2.26^†††^
**Back perception**: FreBAQ-S	11.38 (7.27)	4.98 (4.99)	6.26^**^	1.03^†^
**Low back pain prognosis** (Keele STarT Back Screening Tool, SBST)				
SBST	4.28 (1.98)	–	–	–
SBST, psychosocial	2.44 (1.13)	–	–	–
**Disability**: ODI (%)	23.99 (16.26)	–	–	–
**Fear of movement** (Tampa Scale for Kinesiophobia, TSK)				
TSK	28.07 (6.59)	20.77 (5.62)	7.67^**^	1.19^†††^
TSK, activity avoidance	17.38 (4.59)	12.73 (3.56)	8.05^**^	1.13^†††^
TSK, somatic focus	10.69 (2.93)	8.04 (2.76)	5.51^**^	0.93^†††^
**Catastrophism** (Pain Catastrophizing Scale, PCS)				
PCS	18.09 (11.94)	7.97 (9.18)	5.52^**^	0.95^†††^
PCS, rumination	6.57 (4.33)	3.22 (3.92)	4.9^**^	0.81^†††^
PCS, helplessness	7.5 (5.76)	2.96 (3.84)	5.2^**^	0.93^†††^
PCS, magnification	4.02 (2.64)	1.79 (2.11)	5.49^**^	0.93^†††^
**Depression, anxiety, stress** (Depression, anxiety and stress scale, DASS-21)				
DASS-21	11.88 (11.41)	6.42 (7.97)	3.25^**^	0.55^††^
DASS-21, depression	3.51 (4.1)	1.62 (2.67)	3.61^**^	0.55^††^
DASS-21, anxiety	2.92 (3.78)	1.56 (2.32)	2.66^**^	0.43^††^
DASS-21, stress	5.45 (4.55)	3.23 (3.73)	2.6^**^	0.53^††^
**Avoidance** (Fear-avoidance beliefs questionnaire, FABQ)				
FABQ	38.2 (20.16)	18.72 (14.63)	6.26^**^	1.11^†††^
FABQ, physical activity	13.15 (6.06)	7.19 (6.31)	5.65^**^	0.96^†††^
FABQ, work	16.7 (10.31)	8.69 (8.19)	4.77^**^	0.86^†††^
**Central sensitization**: CSI-A	40.9 (16.79)	–	–	–
**Pain Vigilance and Awareness** (Pain Vigilance and Awareness Questionnaire, PVAQ), mean (SD)				
PVAQ	22.6 (8.31)	13.15 (10.25)	6.17^**^	1.01^†††^
PVAQ, active vigilance	8.16 (4.48)	4.93 (4.35)	4.97^**^	0.73^†††^
PVAQ, passive awareness	14.44 (5.29)	8.22 (6.64)	6.25^**^	1.04^†††^
**Physical activity**: IPAQ	3722.33 (4701.57)	3574.07 (3586.8)	1.66	0.035

CLBP: Chronic Low Back Pain; HC: Healthy controls; SD: standard deviation; Contrast test for continuous variables is *t*-student; for binary/categorical variables is *χ*^2^; Effect size for continuous variables is Cohen’s *d*; for binary/categorical variables is Crammer’s V. FreBAQ-S: Fremantle Back Awareness Questionnaire; ODI: Oswestry Disability Index; CSI-A: Central Sensitization Inventory, Supplementary Appendix A (for CSI-Supplementary Appendix B, see Supplementary material); IPAQ: International Physical Activity Questionnaire. Significance level: ^*^p < 0.05, ^**^p < 0.01. Effect sizes: ^†^small (≥0.2), ^††^medium (≥0.5), ^†††^large (≥0.8).

### 3.1. Cross-cultural adaptation

The translation procedure took 1 month to produce the FreBAQ-S. All the items were easily forward and back-translated. No difficulties were evidenced during the review of the back translations. Some concern was raised with the wording of items 1, 7, 8, and 9, specifically, if the subject should be “the back” (i.e., “My back feels …”) or “the person” (i.e., “I feel my back …”). Following expert recommendations, the translators decided to keep the back as the active subject of the item sentences to keep FreBAQ-S in line with the available translated versions. As no issues emerged from the preliminary testing the researchers confirmed the work done and finalized the FreBAQ-S.

### 3.2. Evidence of validity

The CFA supported the unidimensionality of the FreBAQ-S, even after assessing other potential factor structures (i.e., correlated, and uncorrelated two-factor structures). Two correlated and uncorrelated factor structures suggested in previous studies did not offer a possible solution, as they did not converge. CFA fit indexes supporting a one-factor structure of the questionnaire are reported in Table 2 (*χ*^2^ = 56.17, *p* < 0.001). Additionally, a table including descriptive statistics, skewness, and kurtosis estimates for the items and the total score of the FreBAQ-S, and a figure showing the correlation plot of item correlations after polychoric correlation test are found in Supplementary material (Appendix G). As for the one-factor CFA, both CFI and TLI were above 0.95 (0.97 and 0.96, respectively), supporting a good model fit. Additionally, the SRMR score was 0.07, less than the upper limit of 0.08. Contrary, the RMSEA score of 0.06 was above the recommended value of 0.05. However, we can ensure acceptable adjustment, as it is lower than 0.08 (Hu and Bentler, 1999). SRMRu score was 0.064 suggesting, in line with the other fit indexes, an acceptable fit as it was above 0.08. Estimated parameters of the model are presented in Supplementary material (Appendix H). Additionally, visual inspection of model fit indexes did not support item deletion.

**Table 2:** Confirmatory factor analysis fit indexes.

	Model fit indexes					
CFA (FreBAQ-S)	*X*^2^ (*df*)	RMSEA (CI_90_)	CFI	TLI	SRMR	SRMR_u_
One factor (9 items)	56.17** (27)	0.064 (0.04–0.088)	0.970	0.961	0.067	0.064
One factor (8 items)	42.46** (20)	0.065 (0.038–0.093)	0.973	0.962	0.065	0.063
Two correlated factors						
Two uncorrelated factors						

df: degrees of freedom; RMSEA: Root mean square error of approximation index; CI90: 90% confidence interval; CFI: comparative fit index; TLI: Tucker-Lewis index; SRMR: root mean square of residuals; SRMR_u_: unbiased root mean square of residuals. Although tested, fit measures are not provided for two correlated and two uncorrelated factors because the models did not converge. Significance level: ^*^p < 0.05, ^**^p < 0.01.

Multigroup analysis by health condition and sex as multigroup factors are reported in Table 3. In our sample, a significant decrement in model fit measured by the incremental indexes revealed a lack of measurement invariance. Neither health condition nor sex demonstrated significant influence across the constrained parameters nor significant between-group differences. Mean invariance is not supported by our results, as the incremental indexes (ΔCFI = −0.006 – –0.061; ΔRMSEA = 0.002–0.023; ΔSRMR = 0.005–0.064) exceeded the recommended values (Meredith and Teresi, 2006). According to our results, we can assume that the construct was equally understood between groups and the questionnaire is valid for assessment regardless of sex and clinical status.

**Table 3:** Multigroup confirmatory factor analysis model fit indexes and multigroup comparisons.

	Multigroup CFA model fit indexes						Multigroup comparison		
MI	*X*^2^ (*df*)	RMSEA (CI_90_)	CFI	TLI	SRMR	SRMR_u_	Δ_CFI_	Δ_RMSEA_	Δ_SRMR_
**CLBP and controls**									
**Str**	82.211 (54)	0.052 (0.027–0.073)	0.976	0.968	0.077	0.066			
**Weak**	96.949 (62)	0.054 (0.032–0.074)	0.97	0.965	0.082	0.067	-0.006	0.002	0.005
**Strong**	146.171 (70)	0.075 (0.058–0.092)	0.935	0.933	0.095	0.072	−0.04	0.02	0.01
**Strict**	225.795 (79)	0.098 (0.083–0.113)	0.874	0.885	0.159	0.374	−0.06	0.02	0.06
**Sex differences**									
**Str**	72.400 (54)	0.051 (0.005–0.079)	0.981	0.975	0.074	0.065			
**Weak**	86.657 (62)	0.055 (0.022–0.081)	0.975	0.971	0.081	0.075	−0.006	0.004	0.007
**Strong**	90.826 (70)	0.048 (0.005–0.073)	0.979	0.978	0.082	0.076	−0.004	−0.007	0.001
**Strict**	107.039 (79)	0.052 (0.022–0.076)	0.972	0.974	0.090	0.099	−0.007	0.004	0.008

The regression-invariant model imposes restrictions for parameters (i.e., item loadings, intercepts, and residuals, as well as regression coefficients) to be equal between the clinical condition (Chronic low back pain vs healthy controls) and sex (men vs women) groups. The standardized values of these parameters can vary across groups due to differences in the variances of the observed variables. MI: Model invariance; CLBP: Chronic low back pain; Invar.: invariance; Str: structural – allows the model to freely calculate the parameters for both groups; Weak: forces loadings to be equal between groups; Strong: forces loadings and intercepts to be equal between groups; Strict: forces loadings, intercepts, and residuals to be equal between groups; df: degrees of freedom; RMSEA: Root mean square error of approximation index; CI90: 90% confidence interval; CFI: comparative fit index; TLI: Tucker-Lewis index; SRMR: root mean square of residuals; SRMRu: unbiased root mean square of residuals. Δ_CFI_: incremental comparative fit index; Δ_RMSEA_: incremental root mean square error of approximation index; Δ_SRMR_: incremental root mean square of residuals. Analysis does not support significant differences between tested models.

We found evidence for discriminatory validity, as participants scored significantly higher on the FreBAQ-S than healthy controls. The FreBAQ-S score for the CLBP group (11.38 ± 7.27) was, on average, 6.4 points higher than for the control group (4.98 ± 4.99), with significant between-group differences (*t* = 6.26; *p* < 0.001). No participant achieved the maximum score and only 4.5% of respondents with CLBP scored 0. Although no ceiling effects were found for single items, floor effects were identified for all FreBAQ-S items, while no ceiling nor floor effects were found for summatory FreBAQ-S scores in the CLBP group. A total of 95% of our CLBP participants endorsed some level of impaired self-perception of the back, with only 12 participants scoring zero. All items were endorsed at some level by patients, though with different frequencies. The most strongly endorsed items were 2, 9, and 5, whereas item 3 was the least endorsed.

The correlation analysis supports good convergent validity between the FreBAQ-S and clinical status (see Table 4), as FreBAQ-S scores were correlated with almost all the clinical outcomes evaluated. Positive weak to moderate correlations were found between the FreBAQ-S scores and the following variables: current (*r* = 0.48; *p* < 0.001), usual (*r* = 0.25; *p* < 0.001), and worst pain (*r* = 0.25; *p* < 0.001); kinesiophobia and its subscales; pain catastrophizing and its subscales; depression, anxiety, and stress; fear-avoidance beliefs and its subscales and pain vigilance and awareness and its subscales in both CLBP and healthy samples (0.21 < *r* < 0.48; all *p* < 0.01). Regarding variables only assessed in the CLBP sample, our analysis support significant correlations between prognosis for back pain in SBST total score (*r* = −0.46, *p* < 0.001) and its subscale (*r* = −0.334, *p* < 0.001), disability (*r* = 0.38, *p* < 0.001), and central sensitization (*r* = 0.44, *p* < 0.001). No correlation was found between the FreBAQ-S and the level of physical activity (*p* > 0.48), regardless of the group.

**Table 4:** Correlation analysis between FreBAQ-S scores and standardized variables related to pain experience in the CLBP and HC sample.

	FreBAQ-S	
	CLBP (*N* = 264) Correlation (*p*)	HC (*N* = 128) Correlation (*p*)
**Age**	0.05	0.03
**Weight**	0.08	0.08
**BMI**	0.15^*^	0.13
**Time suffering pain**	−0.001	0.11
**LBP Surgery reception**	−0.17^**^	~
**Current pain**	0.48^**^	0.29^**^
**Usual pain**	0.4^**^	0.46^**^
**Worst pain**	0.25^**^	0.42^**^
**Prognosis (SBST)**	−0.46^**^	~
Prognosis, psychological	−0.33^**^	~
**Disability (ODI)**	0.38^**^	~
**Kinesiophobia (TSK)**	0.37^**^	0.33^**^
TSK, activity avoidance	0.33^**^	0.36^**^
TSK, somatic focus	0.31^**^	0.21^**^
**Catastrophization (PCS)**	0.46^**^	0.35^**^
PCS, rumination	0.37^**^	0.29^**^
PCS, helplessness	0.47^**^	0.34^**^
PCS, magnification	0.43^**^	0.35^**^
**Depression, anxiety, and stress (DASS-21)**	0.49^**^	0.39^**^
DASS-21, depression	0.42^**^	0.35^**^
DASS-21, anxiety	0.43^**^	0.38^**^
DASS-21, stress	0.48^**^	0.34^**^
**Fear-avoidance beliefs (FABQ)**	0.24^**^	0.38^**^
FABQ, physical activity	0.2^**^	0.29^**^
FABQ, work	0.18^**^	0.35^**^
**Central sensitization (CSI, appendix A)**	0.44^**^	~
**Pain vigilance and awareness, PVAQ**	0.26^**^	0.25^**^
PVAQ, active vigilance	0.28^**^	0.29^**^
PVAQ, passive awareness	0.17^**^	0.19^**^
**Physical activity**	0.02	−0.05

Following current guidelines, the strength of the correlations was interpreted as weak (0.1–0.3), moderate (0.3–0.6), and strong (>0.7). CLBP: Chronic low back pain; HC: Healthy controls. Correlations performed with Pearson’s correlation coefficient. ~: not assessed variables at the HC group. ^*^p < 0.05; ^**^p < 0.01.

### 3.3. Evidence of reliability

The FreBAQ-S showed good reliability in terms of Cronbach’s alpha (0.82, 95% CI, 0.79–0.85). We can ensure that there is no duplicity or item redundancy, as Cronbach’s alpha is above 0.7 and does not exceed the value of 0.9 (Streiner, 2010). Test–retest reliability was assessed in CLPB participants (*N* = 113, 41 males, mean 10.8 ± 7.08) and controls (*N* = 45, 13 males, mean 10.8 ± 7.08). For the CLBP sample, the ICC = 0.78 (95%CI: 0.75–0.83) supported acceptable test–retest reliability. The SE was 3.41 and the MDC was 5.12. No significant differences were found between test and test–retest assessments for FreBAQ-S and pain scores in CLBP (*p* = 0.304 and *p* = 0.083) nor healthy (*p* = 0.596 and *p* = 0.428) samples.

## 4. Discussion

The aim of this study was to develop the FreBAQ-S and to evaluate its psychometric properties. The FreBAQ-S was successfully translated and no issues with acceptability, comprehensibility, or relevance were identified. The outcomes of our CFA and MA support the structural validity of the new scale, while the differences found between groups, and the FreBAQ-S positive correlations with clinical status, support its discriminant and convergent validity, respectively. The Cronbach’s alpha scores and test–retest analysis suggest the FreBAQ-S is a reliable questionnaire.

Although previous research supported that the FreBAQ is a unidimensional questionnaire, two correlated and two uncorrelated factor structures were suggested in some of the validation studies (Wand et al., 2016; Janssens et al., 2017; Nishigami et al., 2018). In our study, any purposed two-factor solution did converge. Thus, our results support the unidimensionality of the questionnaire in line with previous reports. Furthermore, our study is, to the best of our knowledge, the first performing multigroup analysis on the FreBAQ, adding novel evidence.

A total of 95% of our CLBP participants endorsed some level of impaired self-perception of the back, supporting a high prevalence of back-disrupted self-perception in our sample. This percentage was similar to previous research (Wand et al., 2014). In line with previously reported floor effects, the percentages of lowest and highest FreBAQ-S scores for the CLBP group were below the cutoff value of 15% that might suggest a possible floor effect (Wand et al., 2016; Janssens et al., 2017; Nishigami et al., 2018; Ehrenbrusthoff et al., 2018b; Erol et al., 2019). This adds confidence to the translation process and the cross-cultural validity of the FreBAQ-S. We also found evidence for discriminatory validity, as participants with CLBP scored significantly higher on the FreBAQ-S than healthy controls, which was consistently supported across different validation studies (Wand et al., 2014;Janssens et al., 2017; Ehrenbrusthoff et al., 2018b; Mahmoudzadeh et al., 2020).

The FreBAQ-S scores were correlated with almost all the clinical outcomes evaluated, partially in line with previous research on the convergent validity of the scale. In this regard, contrary to the German, Dutch, and Persian results (Janssens et al., 2017; Ehrenbrusthoff et al., 2018b; Mahmoudzadeh et al., 2020), we found moderate correlations between FreBAQ-S with current and usual pain, and a weak correlation with worst pain. Although a positive correlation between FreBAQ scores and duration of pain was found for the Turkish and Indian versions (Erol et al., 2019; Rao et al., 2021), our results did not support this correlation, in agreement with other studies assessing the convergent validity of the questionnaire. The moderate correlation found between the FreBAQ-S scores and disability was supported in most of the previous studies (Wand et al., 2016; Janssens et al., 2017; Nishigami et al., 2018; Ehrenbrusthoff et al., 2018b; Erol et al., 2019; Mahmoudzadeh et al., 2020). A possible explanation is that altered back perception might contribute to perceived vulnerability and fitness for purpose of the back, resulting in higher back-related disability (Ehrenbrusthoff et al., 2018b). However, disability was not correlated with the Indian version of the FreBAQ (Rao et al., 2021), suggesting further research on this field.

According to the Fit-For-Purpose Model (Wand, 2022), maladaptive beliefs about the nature of the back problem and future consequences drive behaviors that might bring about maladaptive changes in neurobiological systems that contribute to self-perception of the back. According to that, FreBAQ-S scores would be predicted to be moderately associated with kinesiophobia, pain catastrophizing, depression, anxiety, and stress—consistent with the results found in our study. Briefly, our results agree with current evidence, mostly suggesting that psychological factors are associated with distorted body image (de Moraes Vieira et al., 2014). The correlation between FreBAQ and PCS scores is consistent across all the validations (Wand et al., 2014; Nishigami et al., 2018; Erol et al., 2019; Mahmoudzadeh et al., 2020). However, the Persian and English versions found no relationship with TSK scores (Wand et al., 2014; Mahmoudzadeh et al., 2020). Similar to previous reports, we found positive correlations between FreBAQ and anxiety, depression (Wand et al., 2016; Ehrenbrusthoff et al., 2018b; Erol et al., 2019), and stress (Wand et al., 2016). However, the Persian version found no correlation with psychological status (Mahmoudzadeh et al., 2020), and the Japanese version only supported a correlation with anxiety (Nishigami et al., 2018). Additionally, a weak correlation was found with FABQ and its subscales, like the original English report (Wand et al., 2016).

Unique to this investigation, we explored the associations between FreBAQ-S and central sensitization, prognosis, pain vigilance and awareness, and physical activity. These were assessed following the hypothesis drawn with the Fit-For-Purpose Model (Wand et al., 2022). Evidence-based clinical guidelines consider physical exercise a key component among the nonpharmacological interventions for patients with LBP (Airaksinen et al., 2006; George et al., 2021). However, contrary to our hypothesis, we cannot support a correlation between FreBAQ-S with general physical activity. Previous research on healthy people pointed out that physical activity positively affects body awareness level, in terms of the Body Awareness Questionnaire (Kalkışım et al., 2022). There is also additional evidence of the effects of practicing yoga that facilitates an increase of body awareness (Rivest-Gadbois and Boudrias, 2019). However, those studies were performed on healthy individuals and, consequently, did not use the FreBAQ. In chronic pain, a recent study supports the additional benefit of adding psychomotor therapy to a 12-week group treatment program for increasing body image, especially when applied to participants with low body awareness (van der Maas et al., 2016). Even so, probably due to the novelty of the FreBAQ, our study is, to the best of our knowledge, the first assessing the relation between physical activity and FreBAQ scores. This offers preliminary evidence for encouraging further studies exploring the relationship between body image, pain, and exercise. Furthermore, although previous research suggested a possible relationship between back awareness and poor outcome (Lotze and Moseley, 2007), back awareness does not seem to influence back pain prognosis, as it negatively correlated with the FreBAQ-S (*r* = −0.46, *p* < 0.01). Contrary, according to our predictions, FreBAQ-S was moderately correlated with central sensitization, and weakly with pain vigilance-awareness, suggesting a possible relationship between disrupted body perception and neuroplastic changes (Wand et al., 2016). In this regard, a recent study supported strong inverse correlations between central sensitization and two subscales of the Multidimensional Assessment of Interoceptive Awareness (MAIA) questionnaire (Colgan et al., 2022). Those subscales (Not-Distracting and Not-Worrying) were predictors of lower central sensitization scores in terms of the CSI and lower pain intensity. Furthermore, it was stated that CSI completely mediated the relationship between adaptive body awareness and pain intensity. This correlation between body awareness and central sensitization is maintained in our study when assessing with the FreBAQ-S. Even so, the nature of the study prevents from drawing any inferences of cause and effect between body awareness and the above-mentioned variables. Consequently, they need to be explored in further research.

Consistent with previous validations, we demonstrated an adequate internal consistency of the FreBAQ-S, not affected by item deletion. Thus, the possibility of deleting item 9 suggested in previous studies (Wand et al., 2014; Janssens et al., 2017) is not supported by our results. Additionally, in line with the results previously reported by Ehrenbrusthoff et al. (2018b), our MDC value supported the usability of the FreBAQ-S in clinical and research contexts. This, added to the good level of acceptance, the good comprehensibility, and the adequate time of response of the questionnaire (García-Dopico et al., in press) makes the FreBAQ-S a suitable questionnaire for the assessment of back disrupted body image among Spanish speakers with CLBP.

Overall, our results support the good psychometric properties of the FreBAQ-S. The associations with clinical status offer some support for the clinical relevance of disrupted body perception in CLBP. Body perception disturbance has been related to poor clinical outcomes (Lotze and Moseley, 2007) and psychological distress (Wand et al., 2016), although with some inconsistencies with earlier reports. Those dissimilarities might be explained by sample sizes and pain score differences across studies. However, they should be explored in future research.

Although the adequate psychometric properties of the FreBAQ-S have been demonstrated, there are some limitations that should be considered when interpreting the results of this research. Although all previous validations of the FreBAQ also followed a forward-backward translation procedure, a recent study criticized this methodology (Ozolins et al., 2020). Even considering its inherent limitations, the International Test Commission still considers forward-backward translation as an appropriate design for test adaptation in the ITC Guidelines for Translating and Adapting Tests. The criterion-related validity of the scale is currently unknown (Wand et al., 2014), as there are no gold standard measures of body-perception. The sample was drawn from clinical and non-clinical settings, adding heterogeneity. Although the exclusion criteria were stricter than in previous validations, our sample may not cover the wider CLBP population. The influence of altered self-perception in the development and persistence of CLBP remains uncertain and the nature of the study prevents from drawing any inferences of cause and effect. However, current validations of the FreBAQ were performed on smaller CLBP and control samples (between *N* = 35–104 and *N* = 0–73, respectively) (Wand et al., 2014, 2016; Janssens et al., 2017; Nishigami et al., 2018; Ehrenbrusthoff et al., 2018b; Erol et al., 2019; Mahmoudzadeh et al., 2020). Our sample is larger and meets all requirements for CFA, which increases the statistical significance of our results. Our study is, to our knowledge, the first providing the results of the SRMRu and performing MA on the FreBAQ-S, reinforcing current and adding novel evidence. Additionally, it must be noted that, probably due to the novelty of the questionnaire, current evidence on the use of the FreBAQ is still scarce. Thus, further research is needed to assess the influence of back awareness on CLBP. To cover current research recommendations on CLBP, a mediation analysis of the effect of back awareness among all variables typically related to pain experience could be interesting. In regards to methodological challenges, future lines of research should aim to unravel if the perception of back awareness, explored by the FreBAQ, is equal when assessing different cultures (transcultural studies). In this regard, it would also be interesting to explore the discriminative capacity of the questionnaire in the assessment of different chronic pathologies that associate back pain complaints, such as fibromyalgia. Furthermore, given current evidence of the relation between body awareness, central sensitization, and brain changes in CLBP, specifically in areas related to body awareness, future studies should aim to unravel the relationship between pain, brain changes, and back awareness. This could offer novel insights for assessment and management of CLBP. From our research group, we have also explored patient’s perspectives, assessing what variables influence back awareness in people suffering from CLBP (García-Dopico et al., accepted for publication in Frontiers in Human Neuroscience).

Overall, our results support that the FreBAQ-S, achieved through forward-backward translation, has demonstrated unidimensionality, validity, usability, and reliability to assess disrupted self-perception of the back in Spanish-speaking samples. Further research should assess its influence in the development and persistence of pain.

## Data availability statement

Research data are not shared as are part of an ongoing study. Codes and extended results can be accessed on reasonable request to corresponding author.

## Ethics statement

The studies involving human participants were reviewed and approved by The Clinical Research Ethics Committee of the Balearic Islands (Spain, IB 4502/21 PI). The patients/participants provided their written informed consent to participate in this study.

## Author contributions

NG-D, CS, and OV-R discussed the design of the study. NG-D, CS, OV-R, and BW reviewed the translation process. AT-L, CS, and NG-D guided the statistical analysis. NG-D redacted the manuscript. All authors contributed to the article and approved the submitted version.

## Conflict of interest

The authors declare that the research was conducted in the absence of any commercial or financial relationships that could be construed as a potential conflict of interest.

## Publisher’s note

All claims expressed in this article are solely those of the authors and do not necessarily represent those of their affiliated organizations, or those of the publisher, the editors and the reviewers. Any product that may be evaluated in this article, or claim that may be made by its manufacturer, is not guaranteed or endorsed by the publisher.
