# Supplementary material for: The cross-cultural adaptation, validity, and reliability of the Spanish version of the Fremantle Back Awareness Questionnaire
Source: Front Psychol. 2023 Mar 2;14:1070411. doi: 10.3389/fpsyg.2023.1070411 (PMC10017493; doi:10.3389/fpsyg.2023.1070411)
Supplement: Supplementary file 1 [file Table_1.pdf]

Supplementary Material:

The Cross-Cultural Adaptation, Validity, and Reliability of the Spanish Version of the Fremantle Back Awareness Questionnaire

1 Appendix A. Testing of the pre-final Spanish version of the Fremantle Back Awareness Questionnaire (FreBAQ-S)

The pre-final Spanish version of the Fremantle Back Awareness Questionnaire (FreBAQ-S) was reached by forward backward translation. It was composed of the 9 items included in the original Fremantle Back Awareness Questionnaire (FreBAQ).

This pre-final version was tested in 28 volunteers with CLBP (N=17, 7 males) and without CLBP (N=11, 5 males) between the 10th and the 25th April, 2021. On average, the participants with CLBP aged 39.59 years, and scored 8.53 out of a maximum of 36 points on the preliminary version of the FreBAQ-S. Healthy participants aged, on average, 32.63 years and scored 5.63 points on the FreBAQ-S. Participants of both groups were asked to comment on the acceptability, comprehensibility, and time to complete adequacy of the FreBAQ-S. As any participant gave any improvement suggestion, nor reported any problem regarding the abovementioned aspects, no issues emerged from this step. Thus, the final version of the FreBAQ-S was established (see Supplementary Material, Appendix B). Once the final version of the FreBAQ-S was established, we started the recruitment of participants for the validation study.

2 Appendix B. Fremantle Back Awareness Questionnaire, Spanish Version.

A continuación, se muestran algunas expresiones que otras personas con dolor lumbar utilizan para describir cómo sienten su espalda. Indique si estas frases son aplicables a cómo percibe su espalda cuando experimenta dolor, usando la siguiente escala:

0 = Nunca la siento así.
1 = Raramente la siento así.
2 = Ocasionalmente, o algunas veces la siento así.
3 = Frecuentemente, o una cantidad moderada de tiempo la siento así.
4 = Siempre, o la mayor parte del tiempo la siento así.

Ítems

1. Mi espalda se siente como si no formara parte del resto de mi cuerpo.
2. Necesito enfocar toda mi atención en mi espalda para lograr que se mueva como yo quiero.
3. Siento como si mi espalda se moviera involuntariamente, sin que lo pueda controlar.
4. Cuando realizo mis tareas diarias, no sé cuánto se mueve mi espalda.
5. Cuando realizo mis tareas diarias, no sé exactamente en qué posición se encuentra mi espalda.
6. No soy capaz de percibir el contorno de mi espalda de forma exacta.
7. Mi espalda se siente más grande (hinchada).
8. Mi espalda se siente como si hubiera encogido.
9. Mi espalda se siente como si estuviera torcida (asimétrica).

3 **Appendix C. Frequency of responses to each FreBAQ-S item in the Chronic low back pain patient group (N=264)**

	Response category	Never feels like that	Rarely feels like that	Occasionally or some of the time feels like that	Often, or a moderate amount of time feels like that	Always, or most of the time feels like that	Median	Mean (SD)
	Scoring	0	1	2	3	4		
Item	Frequency of responses	N (%)	N (%)	N (%)	N (%)	N (%)		
1	My back feels as though it is not part of the rest of my body	121 (45.83)	28 (10.60)	51 (19.32)	44 (16.66)	20 (7.57)	1	1.3 (1.39)
2	I need to focus all my attention on my back to make it move the way I want it to	69 (26.14)	52 (19.70)	64 (24.24)	58 (21.97)	21 (7.95)	2	1.66 (1.29)
3	I feel as if my back sometimes moves involuntarily, without my control	167 (63.26)	50 (18.94)	30 (11.36)	13 (4.92)	4 (1.51)	0	0.62 (0.97)
4	When performing everyday tasks, I don’t know how my back is moving	96 (36.36)	64 (24.24)	51 (19.32)	35 (13.25)	18 (6.82)	1	1.3 (1.27)
5	I am not sure exactly what position my back is in	75 (28.40)	63 (23.86)	57 (21.59)	46 (17.42)	23 (8.71)	1	1.54 (1.3)
6	I can’t perceive the exact outline of my back	105 (39.77)	76 (28.78)	37 (1.01)	28 (10.60)	18 (6.81)	1	1.16 (1.25)
7	My back feels like it is enlarged (swollen)	139 (52.65)	46 (17.42)	38 (14.39)	27 (10.22)	14 (5.3)	0	0.98 (1.25)
8	My back feels like it has shrunk	122 (46.21)	51 (19.39)	56 (21.21)	26 (9.85)	9 (3.41)	1	1.05 (1.17)
9	My back feels lopsided (asymmetrical)	72 (27.27)	38 (14.39)	64 (24.24)	60 (22.72)	30 (11.36)	2	1.77 (1.37)

Note: SD: standard deviation.

4 Appendix D. Frequency of responses to each FreBAQ-S item in the healthy control group (N=128)

	Response category	Never feels like that	Rarely feels like that	Occasionally or some of the time feels like that	Often, or a moderate amount of time feels like that	Always, or most of the time feels like that	Median	Mean (SD)
	Scoring	0	1	2	3	4		
Item	Frequency of responses	N (%)	N (%)	N (%)	N (%)	N (%)		
1	My back feels as though it is not part of the rest of my body	95 (74.22)	19 (14.84)	8 (6.25)	2 (1.56)	4 (3.125)	0	0.45 (0.92)
2	I need to focus all my attention on my back to make it move the way I want it to	87 (67.97)	27 (21.09)	10 (7.81)	4 (3.125)	0	0	0.46 (0.77)
3	I feel as if my back sometimes moves involuntarily, without my control	117 (91.41)	9 (7.03)	1 (0.78)	0	1 (0.78)	0	0.12 (0.46)
4	When performing everyday tasks, I don't know how my back is moving	68 (53.125)	31 (24.29)	11 (8.59)	9 (7.03)	9 (7.03)	0	0.91 (1.24)
5	I am not sure exactly what position my back is in	53 (41.41)	36 (28.125)	18 (14.06)	15 (11.72)	6 (4.69)	1	1.1 (1.2)
6	I can't perceive the exact outline of my back	77 (60.16)	37 (28.91)	7 (5.47)	3 (2.34)	4 (3.125)	0	0.59 (0.93)
7	My back feels like it is enlarged (swollen)	107 (83.59)	13 (10.16)	6 (4.69)	2 (1.56)	0	0	0.24 (0.61)
8	My back feels like it has shrunk	100 (78.125)	15 (11.79)	9 (7.03)	4 (3.125)	0	0	0.35 (0.75)
9	My back feels lopsided (asymmetrical)	72 (56.25)	27 (21.09)	21 (16.41)	4 (3.125)	4 (3.125)	0	0.76 (1.04)

Note: SD: standard deviation.

5 **Appendix E. Chronic low back pain participants' history of pain.**

Chronic low back pain (n=264), mean (SD)	
**Self-considered cause(s) of pain (N, %)**	
Non-reported	7 (2.65)
Reported	257 (97.35)
Number of cause(s) reported (N)	318
Work accident	19 (5.97)
Traffic accident	10 (3.14)
Illness	10 (3.14)
Surgery	9 (2.83)
Trauma, fall	18 (5.66)
Spontaneous	174 (54.72)
Sedentarism	4 (1.26)
Labor conditions	8 (2.52)
Stance	11 (3.46)
Scoliosis	7 (2.20)
Hernia	2 (0.63)
Stress / emotional	3 (0.94)
Degeneration	1 (0.31)
Pregnancy and postpartum	7 (2.20)
Sport	8 (2.52)
Overweight	2 (0.63)
Overstrain, overload	10 (3.24)
Environmental conditions (bed…)	2 (0.63)
Vitamin and mineral deficiency	1 (0.31)
Abandonment of sport	1 (0.31)
Nerve pinch	2 (0.63)
Muscle contracture	2 (0.63)
**Diagnosis reception (N, %)**	
Non-received	58 (21.97)
Received	206 (78.03)
Number of different diagnoses received (per person)	2.39 (1.46)
Number of diagnoses reported	555
Non-reported specifical diagnose	59 (10.63)
Low back pain	93 (16.75)
Disc protrusión	58 (10.45)
Herniated disc	68 (12.25)
Muscle contracture	59 (10.63)
Scoliosis	53 (9.55)
Hyperlordosis	15 (2.70)
Sciatica	51 (9.19)

Anterolisthesis / retrolisthesis	3 (0.54)
Canal stenosis	19 (3.42)
Osteoarthritis	42 (7.57)
Autoimmune disease	1 (0.18)
Fibromyalgia	3 (0.54)
Disc degeneration	3 (0.54)
Coccydynia	1 (0.18)
Trochanteritis	1 (0.18)
Chronic pelvic pain	1 (0.18)
Muscle shortening	1 (0.18)
Fracture / Fracture + Wedging	3 (0.54)
Sacral degeneration	1 (0.18)
Hip bursitis	1 (0.18)
Sacroiliitis	1 (0.18)
Vertebral malformation	1 (0.18)
Discopathy	3 (0.54)
Arthritis	1 (0.18)
Radiculopathy	1 (0.18)
Anomaly in coccyx	1 (0.18)
Spondylitis / Ankylosing Spondyloarthritis	1 (0.18)
Inflammation / fibrosis	1 (0.37)
Hip dysmetria	1 (0.37)
Joint hypermobility syndrome	1 (0.37)
Pinch	2 (0.75)
Lumbar sacralization	1 (0.37)
Spondylolysis	1 (0.37)
Spondylolisthesis	1 (0.37)
Spondyloarthritis	1 (0.37)
Lumbar curve rectification	1 (0.37)
Surgeries for low back pain management (N, %)	
No	193 (73.10)
Yes	71 (26.89)
Surgeries received (per person); mean (SD)	1.71 (1.04)
Number of surgeries reported	116
Infiltration	48 (41.38)
Rhizolysis	29 (25)
Epiduroscopy	4 (3.49)
Lumbar decompression surgery	10 (8.62)
Discectomy	7 (6.03)
Laminectomy	7 (6.03)
Spinal / lumbar fusion	5 (4.31)
Radio frequency	1 (0.86)
Phlebography with embolization	1 (0.86)
Hysteroscopies.	1 (0.86)
Epidural block	1 (0.86)
Injected pain reliever	2 (1.72)
Healthcare queries related with pain (mean, SD)	

No	1 (0.38)
Yes	263 (99.62)
Professionals consulted (per person)	2.49 (1.24)
Total number of healthcare queries	652
General practitioner	150 (23.01)
Traumatologist	194 (29.75)
Physical therapist	174 (26.69)
Neurologist	44 (6.75)
Psychologist	19 (2.91)
Osteopath	14 (2.15)
Internist	1 (0.15)
Rheumatologist	12 (1.84)
Pain specialist / unit	9 (1.38)
Neurosurgeon	12 (1.84)
Psiconeuroinmunologist	1 (0.15)
Chiropractor	6 (0.92)
Rehabilitating doctor	2 (0.31)
General surgeon	1 (0.15)
Alternative therapies	1 (0.15)
Urologist	1 (0.15)
Rheumatologist	1 (0.15)
Oncologist	1 (0.15)
Anesthetist	1 (0.15)
Non-reported	8 (1.23)
Central sensitization (Central Sensitization Inventory, CSI), Appendix B; N(%)	
Restless Leg Syndrome	14 (5.3)
Chronic Fatigue Syndrome	12 (4.54)
Fibromyalgia	12 (4.54)
Temporo-mandibular joint pathology	17 (6.43)
Migraine, tension headache	68 (25.75)
Irritable bowel syndrome	28 (10.60)
Multiple chemical sensitivity	1 (0.37)
Cervical injury	86 (32.57)
Anxiety, Panic attack	78 (29.54)
Depression	78 (29.54)

Note: SD: standard deviation.

6 Appendix F. Participants' drug consumption.

	CLBP (n=264)	HC (n=128)	Contrast test	Effect size
Number of drugs per day; mean (SD)	2.63 (1.86)	1.47 (0.76)	2.92**	.82†††
Frequency of drugs consumption per week (N, %)				
Never	82 (31.06)	86 (67.19)	44.48**	.22†
1-2 days	36 (13.64)	7 (5.47)	5.08*	.07
3-4 days	10 (3.79)	0	3.57	.07
>4 days	7 (2.65)	0	2.11	.06
Every day	129 (48.86)	35 (27.34)	15.53**	.13

	CLBP (n=264)	HC (n=128)
Kind of drug consumption (N, %)		
Reported	177 (67.05)	45 (35.16)
Non-reported	87 (32.95)	83 (64.84)
Total drug consumption	484	67
Drugs consumed (N, %)		
Selective proton pump inhibitors	28 (10.61)	7 (5.79)
Inhaled corticosteroid / bronchodil.	7 (2.65)	5 (4.13)
Painkillers	43 (16.28)	6 (4.96)
NSAIDs	77 (29.16)	7 (5.79)
Antipsychotics - neuroleptics	3 (1.13)	0
SSRIss	42 (15.91)	2 (1.65)
Fibrates	1 (0.38)	2 (1.65)
Vitamin D analogs	1 (0.38)	0
Opioids	25 (9.47)	0
Supplements	33 (12.5)	2 (1.65)
Benzodiazepines	22 (8.33)	1 (0.83)
Antiarrhythmics	1 (0.38)	1 (0.83)
Synthetic thyroid hormone	8 (3.03)	3 (2.48)
Antihistamines	8 (3.03)	6 (4.96)
Oral Contraceptives	10 (3.79)	6 (4.96)
Anti-estrogens	0	1 (0.83)
Antihypertensive	23 (8.71)	2 (1.65)
Oral antidiabetics	12 (4.54)	0
Xatin Oxide Inhibitors	1 (0.38)	0
Anti-cholesterolemics	11 (4.16)	2 (1.65)
Alpha blockers	1 (0.38)	0
Antiepileptics / anticonvulsants	17 (6.44)	1 (0.83)
Antivirals	1 (0.38)	0
Anti-Gout	1 (0.38)	0
Enzyme Inhibitors	2 ((0.76)	0
Associatives Calcium-Vitamin D	1 (0.38)	3 (2.48)
Anticoagulants	8 (3.03)	1 (0.83)

Diuretics	5 (1.89)	0
Beta-blockers	9 (3.41)	1 (0.83)
Antidepressants	19 (7.196)	0
Muscle relaxants	7 (2.65)	0
Psychostimulants	0	1 (0.83)
5-Alpha Reductase Inhibitors	1 (0.38)	1 (0.83)
Hormone Replacement Therapy	3 (1.13)	0
Antispasmodics	1 (0.38)	0
Adrenergic beta-agonists	1 (0.38)	0
ACE Inhibitors	9 (3.41)	1 (0.83)
Antibiotics	3 (1.13)	0
Dopamine Agonists	0	0
Statins	11 (4.16)	3 (2.48)
Progestins	1 (0.38)	0
Antacid	1 (0.38)	0
Immunosuppressive Drugs	3 (1.14)	0
Non-NSAID Anti-inflammatory	2 (0.76)	0
Leukotriene receptor antagonist	4 (1.51)	0
Antifibrinolytic	1 (0.38)	0
Bisphosphonates	1 (0.38)	1 (0.83)
Alpha-1 agonist	1 (0.38)	0
Triptans	0	1 (0.83)
Narcotic Pain Relievers	1 (0.38)	0
Antimalarial	1 (0.38)	0
Antithyroid	1 (0.38)	0
Oral Bronchodilators	2 (0.75)	0
Alpha and beta receptor blockers	0	0
Mucolytics	1 (0.38)	0
Antimuscarinics	1 (0.38)	0
Anxiolytics	2 (0.75)	0
Injected antidiabetics	1 (0.38)	0
Propulsion drugs	1 (0.38)	0
Drugs for Addictive Disorders	1 (0.38)	0
Cough suppressant	1 (0.38)	0
Antiemetic	1 (0.38)	0

Note: CLBP: Chronic Low Back Pain; HC: Healthy controls; Contrast test for continuous variables is t-student; for binary/categorical variables is χ^2; Effect size for continuous variables is Cohen's d; for binary/categorical variables is Crammer's V. NSAIDs: Non-steroideal antiinflamatory drugs;

*SSRIs: Selective serotonin reuptake inhibitor; ACE: Angiotensin Converting Enzyme. Significance level: *p<.05, **p<.01. Effect sizes: small (≥0.2)†, medium (≥0.5)††, large (≥0.8)†††*

7 Appendix G. Descriptive statistics, skewness and kurtosis for single items and total scores for the FreBAQ-S and correlation plot of item correlations.

**Item**	***M***	***SD***	**Skewness**	**Kurtosis**
1	1.02	1.31	0.93	-0.52
2	1.27	1.28	0.56	-0.94
3	0.46	0.87	**2.04**	**3.71**
4	1.17	1.27	0.81	-0.51
5	1.4	1.29	0.51	-0.91
6	0.97	1.18	1.15	0.35
7	0.74	1.14	1.4	0.85
8	0.82	1.1	1.11	0.14
9	1.44	1.35	0.41	-1.12
Total	9.08	7.31	0.89	0.7

	i1	i2	i3	i4	i5	i6	i7	i8	i9
i1	1.00	0.66	0.65	0.39	0.35	0.44	0.46	0.39	0.29
i2		1.00	0.73	0.41	0.38	0.52	0.56	0.50	0.45
i3			1.00	0.53	0.50	0.57	0.53	0.47	0.44
i4				1.00	0.77	0.60	0.45	0.38	0.34
i5					1.00	0.66	0.37	0.37	0.38
i6						1.00	0.48	0.49	0.41
i7							1.00	0.49	0.49
i8								1.00	0.54
i9									1.00

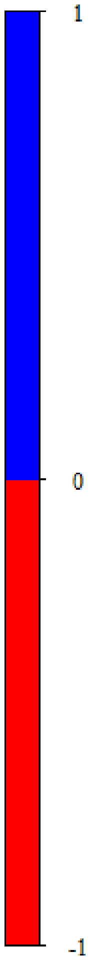

8 Appendix H. Estimated parameters for FreBAQ-S one-factor CFA on the chronic low back pain sample

Parameter	Estimate	*SE*	*Z*	Standarized
Latent variables				
FreBAQ-S (Item 1)	1.000			
FreBAQ-S (Item 2)	1.096	0.112	9.759	0.000
FreBAQ-S (Item 3)	0.818	0.087	9.457	0.000
FreBAQ-S (Item 4)	1.115	0.115	9.692	0.000
FreBAQ-S (Item 5)	1.130	0.115	9.818	0.000
FreBAQ-S (Item 6)	1.159	0.117	9.880	0.000
FreBAQ-S (Item 7)	0.876	0.100	8.786	0.000
FreBAQ-S (Item 8)	0.756	0.087	8.654	0.000
FreBAQ-S (Item 9)	0.817	0.092	8.832	0.000
Intercepts				
FreBAQ-S (Item 1)	1.295	0.085	15.191	0.000
FreBAQ-S (Item 2)	1.659	0.080	20.857	0.000
FreBAQ-S (Item 3)	0.625	0.060	10.460	0.000
FreBAQ-S (Item 4)	1.299	0.078	16.599	0.000
FreBAQ-S (Item 5)	1.542	0.080	19.245	0.000
FreBAQ-S (Item 6)	1.159	0.077	15.096	0.000
FreBAQ-S (Item 7)	0.981	0.077	12.753	0.000
FreBAQ-S (Item 8)	1.049	0.072	14.524	0.000
FreBAQ-S (Item 9)	1.765	0.084	20.981	0.000
FreBAQ-S	0.000	-	-	-
Variances				
FreBAQ-S (Item 1)	1.351	0.139	9.736	0.000
FreBAQ-S (Item 2)	0.986	0.129	7.663	0.000
FreBAQ-S (Item 3)	0.561	0.122	4.596	0.000
FreBAQ-S (Item 4)	0.909	0.144	6.297	0.000
FreBAQ-S (Item 5)	0.967	0.138	6.987	0.000
FreBAQ-S (Item 6)	0.791	0.157	5.042	0.000
FreBAQ-S (Item 7)	1.126	0.147	7.675	0.000
FreBAQ-S (Item 8)	1.053	0.118	8.933	0.000
FreBAQ-S (Item 9)	1.489	0.117	12.697	0.000
FreBAQ-S	0.569	0.085	6.659	
R-Square				
FreBAQ-S (Item 1)	0.296	-	-	-
FreBAQ-S (Item 2)	0.409	-	-	-
FreBAQ-S (Item 3)	0.405	-	-	-
FreBAQ-S (Item 4)	0.438	-	-	-
FreBAQ-S (Item 5)	0.429	-	-	-
FreBAQ-S (Item 6)	0.492	-	-	-
FreBAQ-S (Item 7)	0.279	-	-	-

FreBAQ-S (Item 8)	0.236	-	-	-
FreBAQ-S (Item 9)	0.203	-	-	-

Note: SE = FreBAQ-S = Fremantle Back Awareness Questionnaire, Spanish version.

Standard error. Z = Z-based contrast test statistic. Standardized = Standardized coefficient. Item 1 is fixed to identify the model.
